# Endoscopic ultrasound-guided side-fenestrated needle biopsy sampling is sensitive for pancreatic neuroendocrine tumors but inadequate for tumor grading: a prospective study

**DOI:** 10.1038/s41598-022-09923-1

**Published:** 2022-04-08

**Authors:** Alexander Appelstrand, Fredrik Bergstedt, Anna-Karin Elf, Henrik Fagman, Per Hedenström

**Affiliations:** 1 Department of Clinical Pathology, Sahlgrenska University Hospital, Gothenburg, Sweden; 2 Department of Surgery, Sahlgrenska University Hospital, Gothenburg, Sweden; 3 Sahlgrenska Center for Cancer Research, Department of Laboratory Medicine, Institute of Biomedicine, Sahlgrenska Academy at University of Gothenburg, Gothenburg, Sweden; 4 Department of Molecular and Clinical Medicine, Institute of Medicine, Sahlgrenska Academy at University of Gothenburg, Gothenburg, Sweden; 5 Division of Medical Gastroenterology, Department of Internal Medicine, Sahlgrenska University Hospital, Medicinmottagningen, Sahlgrenska Sjukhuset, Blå Stråket 3, 413 35 Gothenburg, Sweden

**Keywords:** Endocrine cancer, Gastroenterology

## Abstract

Accurate pretreatment grading of pancreatic neuroendocrine tumors (PanNETs) is important to guide patient management. We aimed to evaluate endoscopic ultrasound-guided fine needle biopsy sampling (EUS-FNB) for the preoperative diagnosis and grading of PanNETs. In a tertiary-center setting, patients with suspected PanNETs were prospectively subjected to 22-gauge, reverse-bevel EUS-FNB. The EUS-FNB samples (Ki-67_EUS_) and corresponding surgical specimens (Ki-67_SURG_) were analyzed with Ki-67 indexing and thereafter tumor grading, (GRADE_EUS_) and (GRADE_SURG_) respectively. In total 52 PanNET-patients [median age: 66 years; females: 25/52; surgical resection 22/52 (42%)] were included. EUS-FNB was diagnostic in 44/52 (85%). In 42 available FNB-slides, the median neoplastic cell count was 1034 (IQR: 504–3667) with 32/42 (76%), 22/42 (52%), and 14/42 (33%) cases exceeding 500, 1000, and 2000 neoplastic cells respectively. Ki-67_SURG_ was significantly higher compared to Ki-67_EUS_ with a moderate correlation comparing Ki-67_EUS_ and Ki-67_SURG_ (Pearson r = 0.60, r^2^ = 0.36, p = 0.011). The GRADE_EUS_ had a weak level of agreement (κ = 0.08) compared with GRADE_SURG_. Only 2/12 (17%) G2-tumors were correctly graded in EUS-FNB-samples. EUS-guided fine needle biopsy sampling is sensitive for preoperative diagnosis of PanNET but biopsy quality is relatively poor. Therefore, the approach seems suboptimal for pretreatment grading of PanNET.

## Introduction

While pancreatic neuroendocrine tumors (PanNETs) constitute a minority of all pancreatic neoplasms^1^, patient long-term survival in PanNET is significantly higher as compared with pancreatic adenocarcinoma^2^. Nevertheless, the individual prognosis in patients suffering from PanNET is much dependent on the tumor characteristics in general and the tumor grade in particular^3^.

PanNETs are classified according to the the New World Health Organization Classification for Pancreatic Neuroendocrine Neoplasia^4^. PanNET tumor grade (G1-G3) is based on the tumor proliferation rate as measured by the Ki-67 Index^4,5^. Accurate determination of the Ki-67 Index is important with respect to the prognosis^6,7^. Therapeutic options of PanNET includes surgery, somatostatin analogues, peptide receptor radionuclide therapy, molecular targeted treatment, and chemotherapy with the approach being determined upon tumor size, stage, and location^8^. Tumor grade may also influence the choice of therapy, especially in elderly patients with comorbidities and with a high risk–benefit ratio in pancreatic surgery.

To determine the Ki-67 Index, it is recommended that a minimum of 500 neoplastic cells, preferably 2000 neoplastic cells, should be identified and counted in the tumor sample^9^. Manual counting of Ki-67 positive cells is considered an accurate, but time consuming, method for the estimation of the Ki-67 Index in PanNETs^10^. Digital quantification of the Ki-67 Index in neuroendocrine tumors has emerged as an alternative and reliable method^11^.

Endoscopic ultrasound (EUS) is a highly sensitive imaging modality for the identification of small pancreatic lesions such as PanNETs^12–14^. Moreover, EUS-guided fine-needle aspiration (EUS-FNA) is accurate for the diagnosis of unclear pancreatic lesions by cytopathology^15^. Even though the sensitivity of EUS-FNA for PanNETs is reportedly as high as ~ 90%^16^, EUS-FNA aspirates cannot provide tissue specimens for histology and the FNA-yield is often insufficient for determination of the Ki-67 Index^17^. In recent years, the development of new needles intended for EUS-guided fine needle biopsy sampling (EUS-FNB) with a side-fenestrated design^18^ or end-cutting design^19^ has facilitated the acquisition of tissue cores and thereby the diagnosis of neoplasms such as mesenchymal tumors^20^. However, it is poorly studied if EUS-FNB using a side-fenestrated needle may provide tissue cores that are sufficient for accurate Ki-67-indexing, and thereby grading, of pretreatment PanNETs.

To summarize, the reliable assessment of the Ki-67 Index in PanNETs already at the preoperative stage is of great importance to determine the prognosis and to adequately select patients with respect to surgical resection. The aim of the current study was to investigate if EUS-FNB using a side-fenestrated needle is sensitive for PanNET and if pretreatment quantification of the Ki-67 Index in EUS-FNB biopsy samples is accurate for the preoperative grading of PanNETs.

## Methods

### Study setting and study population

Patients aged > 18 years referred to the Sahlgrenska university hospital (SUH) tertiary center endoscopy unit during 2015–2019 based on clinical suspicion of a PanNET for diagnostic EUS were eligible for consecutive enrolment in this prospective, single-centre study. Patients were excluded if no EUS-FNB was performed or the final diagnosis turned out not to be PanNET. Written informed consent was obtained from all study subjects. The STARD guidelines for reporting diagnostic studies were applied in the current work.

The study was approved by the Regional Ethical Review Board of Gothenburg (Dnr:1092-11). All methods were performed in accordance with the relevant guidelines and regulations. The study was registered at ClinicalTrials.gov (11/02/2015) with study registration number (NCT02360839).

### The EUS-procedure

A linear echoendoscope (EG3870UTK, Pentax, Japan) and an ultrasound processor (HI VISON Ascendus, Hitachi, Tokyo, Japan) were used to examine the patients under deep sedation. The characteristics of target lesions were recorded. Before sampling, the echoendoscope was stabilized in the stomach or in the duodenum. Then, transmural puncture of the target lesion was performed by EUS-FNB using a 22 gauge reverse-bevel needle (EchoTip Procore®, Wilson-Cook Medical, Limerick, Ireland) and by applying fanning and standard suction^21^. All EUS-procedures of the study were performed by either of two dedicated and experienced endosonographers (> 1000 procedures).

The yield of EUS-FNB was put into formalin tubes and the FNB-core was assessed macroscopically. Additional FNB-passes were performed if the cores were considered inadequate at gross examination. No fixed number of passes was performed. Routine EUS-FNA (EchoTip®, Wilson-Cook Medical), and not EUS-FNB, was preferred during some periods when diagnostics was performed by subspecialized cytopathologists or if no FNB-needle was available on-site.

### Histopathology

First, FNB-core biopsy samples were formalin-fixed and paraffin-embedded (FFPE) as per standard protocols. Sections (3‒4 µm) were placed on positively charged glass slides and antigen retrieval performed using the Dako PT-Link system, using EnVision™ FLEX Target Retrieval Solution (TRS High). Samples were routinely stained with hematoxylin–eosin and immunohistochemistry was performed using the Dako Autostainer Link using EnVision™ FLEX according to the manufacturer’s instructions (DakoCytomation).

The FNB-samples were regarded diagnostic for PanNET only if cytomorphology and immunohistochemistry [positive staining for chromogranine A (CGA) and synaptophysin (SYN)] were consistent with the diagnosis. Else, samples were regarded non-diagnostic for PanNET.

### Quantification of neoplastic cells and Ki-67-indexing

Haematoxylin/eosin (H&E), synaptophysin (SYN) and Ki67-stained slides were scanned on a NanoZoomer S210 (Hamamatsu, Hamamatsu City, Japan) or an Oncotopix Scan (Visiopharm, Copenhagen, Denmark) using the NanoZoomer Digital Pathology (NDP) Scan v. 3.2.15 (Hamamatsu) software. Scanning was performed at 40 × magnification setting with manual definition of focus points (20–30 (HE and SYN slides) or 50–100 (Ki67 slides) focus points were evenly distributed, depending on the size of the sample). Parallel H&E, Ki67 and SYN immunohistochemically stained slides were viewed synchronously in the digital viewer [Visiopharm Oncotopix (version 2018.4.6.4744)] to locate neoplastic cells (SYN +) on the Ki67 stained slides and to guide selection of areas of interest (AOI) for digital quantification.

Areas of interest (AOI) for digital quantification of the EUS-FNB samples were defined manually to exclude regions with appreciable presence of lymphocytes. Only groups of more than five neoplastic cells were selected, to avoid counting other cell types such as lymphocytes, granulocytes, fibroblasts or ordinary pancreatic glandular cells that could not be unambiguously differentiated from dissociated neoplastic cells. If a sufficient number of neoplastic cells were not present in the first AOI, a secondary hotspot was located and an additional AOI was manually defined to obtain a maximum amount of neoplastic cells counted (preferably 2000 cells). AOI in the resection specimens were defined as a circular region with a diameter of 500 µm drawn around the region of the highest fraction of Ki67 positivity (proliferative hotspot) determined in the 10 × magnification of the app.

Digital quantification of the total number of neoplastic cells in the AOIs and the fraction of Ki67 positive cell nuclei was performed using the #10143 Ki-67 Neuroendocrine Neoplasm app for Oncotopix from Visiopharm, using original app settings as provided by the manufacturer. For EUS-FNB samples, the total number of neoplastic cells was counted on Ki-67 slides and documented for the three largest groups of neoplastic cells (Cell count_EUS_). For comparison, the total number of neoplastic cells was also counted in the ten largest groups of neoplastic cells.

Finally, the Ki-67 Index (%) of EUS-FNB samples (Ki-67_EUS_) was determined by dividing the number of positive cells by the total number of cells counted in the three largest groups of neoplastic cells. Based on the Ki-67_EUS_, the tumor grade (GRADE_EUS_) was estimated. For resection specimens, areas of non-neoplastic tissue (e.g. connective tissue and erythrocytes) were digitally removed by the tumor detection-algorithm of the app. DAB-positivity was scored as above and a minimum of 2000 neoplastic cells were counted in every specimen.

The Ki-67 Index (%) of resection specimens (Ki-67_SURG_) was determined by dividing the number of positive cells by the total number of cells counted. Based on the Ki-67_SURG_, the tumor grade (GRADE_SURG_) was determined.

Regarding both EUS-FNB samples and resection specimens, the Ki-67 Index (%) determined as per standard diagnostic practice (manual cell counting in microscope and on printed screenshots) were available for comparison. The pathologist performing the quantification of the resection specimens was blinded to the quantification of EUS-FNB samples.

The WHO classification of 2017^4^ was applied for tumor grading. In study cases handled before 2017, the WHO classification of 2010^22,23^ was applied but with a similar and pragmatic management as the 2017 version regarding the distinction between G1 and G2 of tumors with a Ki-67 Index above 2 but less than 3, Table 2.

### Clinical follow up including patient management and surgery

All study patients were monitored via clinical follow-up for a minimum of six months and, if needed, for an extended period at least until the final diagnosis was established. The management of the study subjects was determined at the local multi-disciplinary therapy conference based on international guidelines^4,6,24^. Final diagnosis was based on the surgical specimen. In patients not subjected to surgery, the combination of clinical follow-up including biochemistry, radiology, somatostatin receptor imaging, and any other sampling modality for pathology was used as the reference standard. Cases with an unclear final diagnosis were not regarded as PanNET.

### Study outcomes

The outcomes of this study were:

- The sensitivity of EUS-FNB for PanNET.
- The EUS-FNB-biopsy quality, i.e. the Cell Count_EUS_.

The cut-off level for a poor, fair, good, and excellent biopsy quality was set at < 500, 500–1000, 1000–2000, and > 2000 neoplastic cells respectively. All non-diagnostic EUS-FNB samples were per definition < 500 neoplastic cells.

- The accuracy of pretreatment Ki-67 indexing (Ki-67_EUS_) and PanNET tumor grading (GRADE_EUS_) in EUS-FNB samples as compared with Ki-67 indexing (Ki-67_SURG_) and grading (GRADE_SURG_) in surgical specimens.

### Statistical analysis

Descriptive, continuous data were described as median and interquartile range (IQR), while descriptive, categorical data were described as frequencies.

In the calculation of the diagnostic sensitivity of EUS-FNB, an intention-to-diagnose analysis was performed.

Fisher’s exact test was used in the proportional analysis of any factors with a potential impact on the sampling yield and biopsy quality of EUS-FNB.

Pearson’s test was used to calculate the correlation coefficient (r-value) between the Ki-67 Index as in EUS-FNB samples and as in surgical specimens. Additionally, Wilcoxon signed rank test was used to identify any difference in the Ki-67 Index comparing EUS-FNB samples and their corresponding surgical specimens.

Cohen’s kappa value was calculated to describe the level of agreement comparing PanNET grading in EUS-FNB samples and PanNET grading in the corresponding surgical specimens. Both an intention-to-diagnose analysis and a per-protocol analysis was performed.

A p-value of < 0.05 was considered statistically significant in all analyses. The statistical calculations and tests were performed using IBM SPSS Statistics version 25.0.

## Results

**Figure 1:**
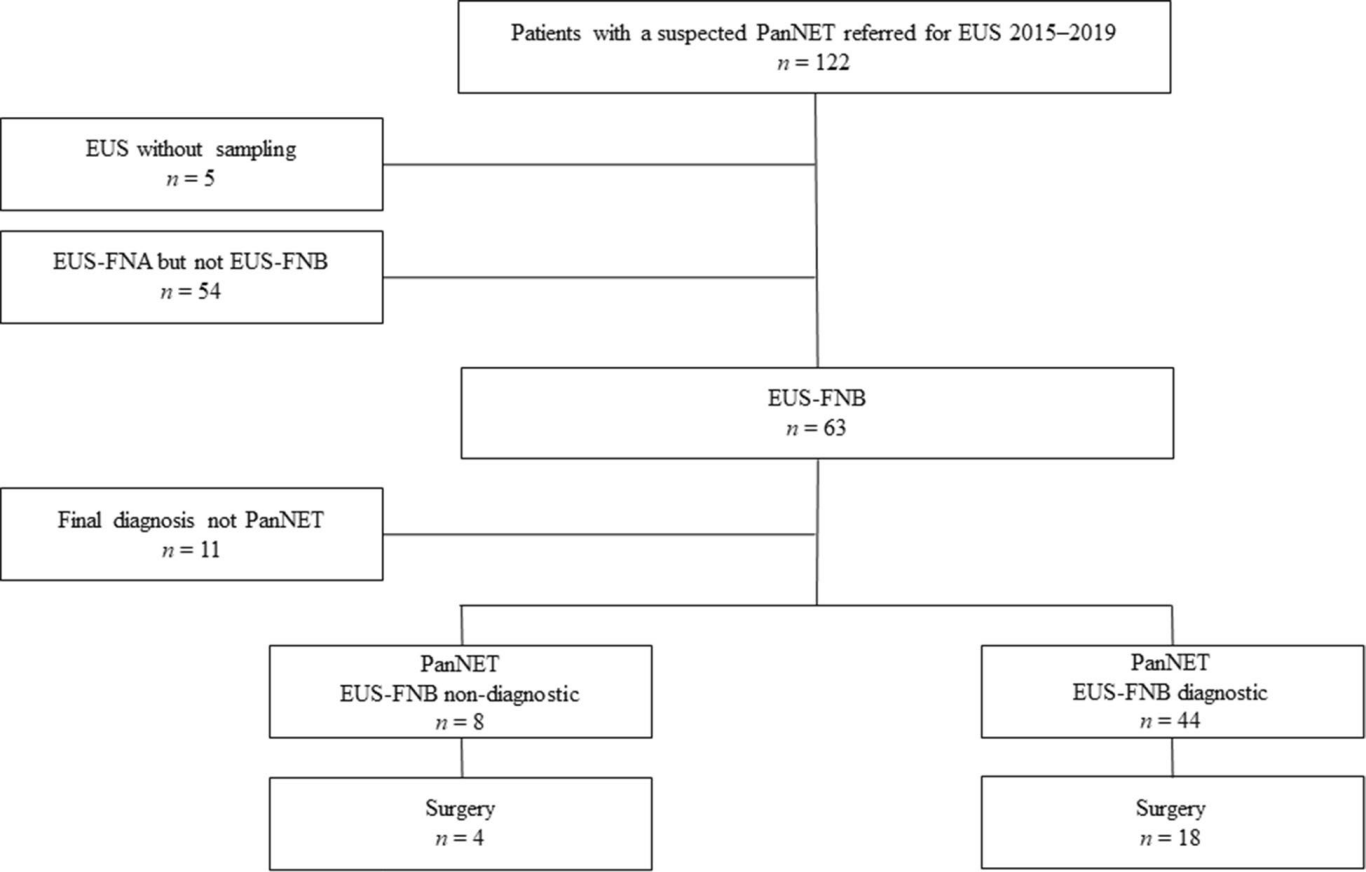
A flow chart of the enrolment process of the study cases. *PanNET* pancreatic neuroendocrine tumors, *EUS-FNB* endoscopic ultrasound-guided fine-needle biopsy sampling.

In total 52 patients with PanNET (median age: 66 years; females: 25/52) were subjected to EUS-FNB and finally included in the study, Fig. 1.

Surgical resection of the tumor within the study follow-up time frame was performed in 22/52 (42%) patients. None of these 22 patients were treated with neoadjuvant therapy before surgery. In the remaining 30 patients, the reason for not performing surgery was: metastatic PanNET ± comorbidity n = 12; small PanNET with surveillance only n = 10; comorbidity n = 4; MEN-1 with surveillance only n = 3; awaiting surgery n = 1. The study base-line characteristics were as presented in Table 1.

**Table 1:** The study baseline characteristics.

	All cases (n = 52)	Cases resected (n = 22)
**Patient characteristics**		
Age, median (IQR)	66 (53–72)	60 (50–70)
Female gender, n (%)	25 (48)	10 (43)
**Tumor characteristics**		
Tumor size at CT scan (mm), median (IQR)	25 (15–52)	30 (19–50)
Tumor position (head/neck/body/tail)	20/2/15/15	10/0/5/7
Tumor character (solid/semisolid/cystic)	35/15/2	14/8/0
Tumor type^a^ (non-functioning/functioning)	50/2	20/2
Tumor grade^b^ (G1/G2/G3)	–	10/12/0
Follow-up (months), median (IQR)	26 (10–55)	38 (14–56)
**EUS procedure characteristics**		
Size at EUS (mm), median (IQR)	24 (14–44)	30 (20–40)
Sampling route (transduodenal/transgastric)	20/32	9/13
Number of needle passes, (1/2/3), median (IQR)	15/27/10, 2 (1–2)	7/10/5, 2 (1–2)

^a^Tumor type was based on clinical symptoms caused by tumor hormone production. Both patients with a functioning tumor had an insulinoma.

^b^Based upon the Ki-67 Index calculated in the resection specimens (n = 22).

### Study outcomes

#### The sensitivity of EUS-FNB for PanNET

Pathology of EUS-FNB samples was diagnostic for PanNET in 44/52 (85%) of all patients and in 18/22 (82%) of patients subjected to surgery during the study time frame, Fig. 1.

#### The biopsy quality and neoplastic cell count in EUS-FNB samples

In 42/44 (95%) of the diagnostic EUS-FNB samples, digital quantification of neoplastic cells was performed. In one case (#22, not subjected to surgery), the EUS-FNB sample was accidentally lost during the process and in the other case (#34, subjected to surgery) no quantification could be performed due to staining artifacts.

Including all the above 42 patients (22 males and 20 females), the median Cell Count_EUS_ was 1034 (IQR: 504–3667) with 32/42 (76%), 22/42 (52%), and 14/42 (33%) cases exceeding 500, 1000, and 2000 neoplastic cells respectively, Table 2. Including only patients subjected to surgery (n = 17; 10 males and 7 females), the median Cell Count_EUS_ was 959 (IQR: 497–1814) with 13/17 (76%), 7/17 (41%), and 3/17 (18%) cases exceeding 500, 1000, and 2000 neoplastic cells respectively, Table 2.

**Table 2:** The Ki-67 Index and tumor grade in EUS-FNB samples and resection specimens.

Case #	EUS-FNB samples				Resection specimens	
	Ki-67-positive cells (n)	Neoplastic cells (n)	Ki-67 Index (%)	GRADE (G1/G2/G3)	Ki-67 Index (%)	GRADE (G1/G2/G3)
1	1872	4692	39.9	3	No surgery	No surgery
2	2	259	0.8	1	No surgery	No surgery
3	1	717	0.1	1	No surgery	No surgery
4	3026	10,216	29.6	3	No surgery	No surgery
5	1	524	0.2	1	No surgery	No surgery
6	0	109	0	1	6.3	2
7	12	1034	1.2	1	No surgery	No surgery
8	785	2834	27.7	3	No surgery	No surgery
9	64	518	12.4	2	15.5	2
10	17	959	1.8	1	1.8	1
11	389	8102	4.8	2	No surgery	No surgery
12	9	873	1	1	3.7	2
13	2	1277	0.2	1	1.5	1
14	0	489	0	1	No surgery	No surgery
15	1	628	0.2	1	No surgery	No surgery
16	27	978	2.8	1	2.9	1
17	1	476	0.2	1	9.4	2
18	18	342	5.3	2	No surgery	No surgery
19	66	5365	1.2	1	No surgery	No surgery
20	80	1597	5	2	4.2	2
21	8	614	1,3	1	6.1	2
22	Glass lost	Glass lost	NA	NA	No surgery	No surgery
23	FNB non-dia	FNB non-dia	NA	NA	No surgery	No surgery
24	FNB non-dia	FNB non-dia	NA	NA	2.2	1
25	97	2008	4.8	2	No surgery	No surgery
26	FNB non-dia	FNB non-dia	NA	NA	No surgery	No surgery
27	FNB non-dia	FNB non-dia	NA	NA	2.4	1
28	FNB non-dia	FNB non-dia	NA	NA	2.4	1
29	95	469	20.3	3	No surgery	No surgery
30	9	376	2.4	1	No surgery	No surgery
31	2	472	0.4	1	9.4	2
32	FNB non-dia	FNB non-dia	NA	NA	5.9	2
33	0	97	0	1	No surgery	No surgery
34	Artifacts	Artifacts	NA	NA	9.4	2
35	6	1638	0.4	1	0.9	1
36	89	4273	2.1	1	1.3	1
37	399	4796	8.3	2	No surgery	No surgery
38	38	2857	1.3	1	6.4	2
39	FNB non-dia	FNB non-dia	NA	NA	No surgery	No surgery
40	484	32,061	1.5	1	3.5	2
41	0	51	0	1	No surgery	No surgery
42	13	446	2.9	1	6.4	2
43*	15	1990	0.8	1	1.9	1
44	FNB non-dia	FNB non-dia	NA	NA	No surgery	No surgery
45*	3	3060	0.1	1	No surgery	No surgery
46	2	689	0.3	1	No surgery	No surgery
47	11	558	2	1	2.8	1
48	322	12,123	2.7	1	No surgery	No surgery
49	10	1066	0.9	1	No surgery	No surgery
50	90	9620	0.9	1	No surgery	No surgery
51	5396	13,559	39.8	3	No surgery	No surgery
52	3	1728	0.2	1	No surgery	No surgery

*Cases presenting as a cystic PanNET with only a sparse solid component.

No tested factor had a significant impact on the biopsy yield and quality in EUS-FNB samples, Table 3.

**Table 3:** Factors with a potential impact on the biopsy yield and quality of EUS-FNB samples.

	FNB sample diagnostic, n/n_tot_ (%)	p-value	Cell Count_EUS_ > 1000, n/n_tot_ (%)	p-value
**Tumor size**		0.13		1.0
≤ 20 mm	19/25 (76)		10/25 (40)	
> 20 mm	25/27 (93)		11/27 (41)	
**Tumor type**		1.0		1.0
Solid	30/35 (86)		14/35 (40)	
Cystic^a^	14/17 (82)		7/17 (41)	
**Tumor position**		1.0		0.26
Head	17/20 (85)		6/20 (30)	
Body-tail	27/32 (84)		15/32 (47)	
**FNB-needle passes**		1.0		0.10
1–2 passes	35/42 (83)		14/42 (33)	
3 passes or more	9/10 (90)		7/11 (64)	

^a^Tumor with a partially or entirely cystic appearance at endosonography.

#### Accuracy of Ki-67-indexing and grading of PanNET in EUS-FNB samples

Quantification and assessment of the Ki-67 Index in EUS-FNB samples (Ki-67_EUS_) was according to Table 2.

**Figure 2:**
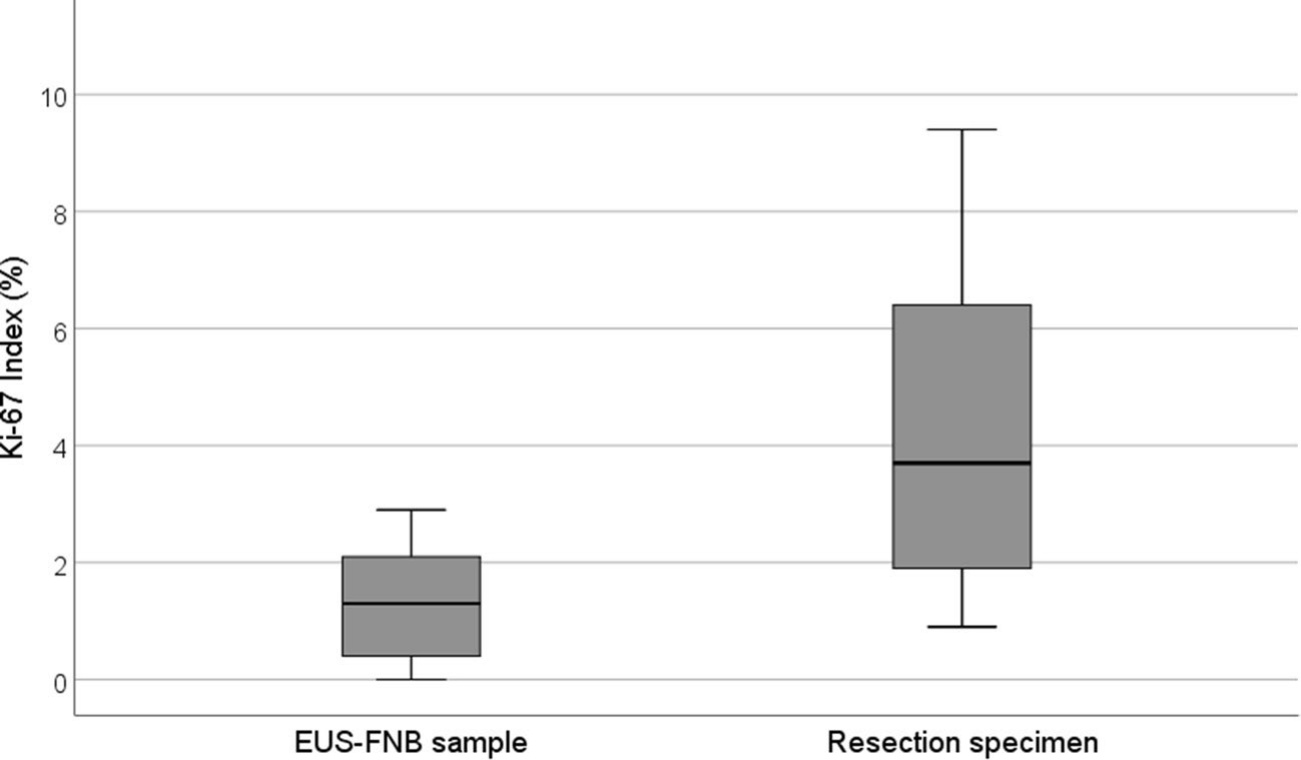
A box-plot of study cases subjected to surgical resection (n = 17) showing the estimated Ki-67 Index calculated in the EUS-FNB samples and the reference Ki-67 Index calculated in the corresponding resection specimens. The bold line signifies the median, the box signifies the IQR, and the T-bars signify the 95% confidence interval. The five resected cases without a corresponding, diagnostic EUS-FNB sample available for Ki-67-indexing were excluded from the plot.

In the 17 cases which were subjected to surgery (#34 not included due to staining artifacts), there was only a moderate correlation comparing the Ki-67_EUS_ and the Ki-67_SURG_ (Pearson r = 0.60, r^2^ = 0.36, p = 0.011). The Ki-67_SURG_ was significantly higher compared the Ki-67_EUS_, Fig. 2.

Based on the Ki-67_EUS_, pretreatment tumor grading of the study PanNETs (GRADE_EUS_) was according to Table 2. The GRADE_EUS_ was found to have a weak level of agreement as compared with the tumor grade in the corresponding resection specimens (GRADE_SURG_), Table 4. In the intention-to-treat analysis, only 2/12 (17%) tumors graded as G2 in surgical specimens (GRADE_SURG_) were indeed correctly graded as G2 also in EUS-FNB samples (GRADE_EUS_). Analyzing only the cases with a Cell Count_EUS_ > 1000 cells (n = 7: G1 n = 4; G2 n = 3), still 2/3 (67%) G2-tumors (GRADE_SURG_) were graded as G1-tumors in FNB biopsy samples (GRADE_EUS_).

**Table 4:** The tumor grade of resected PanNETs (n = 22) as assessed in EUS-FNB samples (GRADE_EUS_) and in the corresponding surgical specimens (GRADE_SURG_).

GRADE_EUS_	GRADE_SURG_		
	G1	G2	G3
G1	7	8	0
G2	0	2	0
G3	0	0	0
Non-diagnostic FNB^a^	3	2	0

^a^EUS-FNB samples being non-diagnostic at pathology including immunohistochemistry.

For comparison, the quantification of neoplastic cells and assessment of the Ki-67 Index in the ten largest groups of neoplastic cells in the EUS-FNB samples did not result in a significant change in outcome, Supplementary Table 1 in Supplementary Materials.

## Discussion

In this prospective study we have shown that EUS-guided fine needle biopsy sampling, using a 22-gauge reverse bevel needle, is sensitive for the diagnosis of pancreatic neuroendocrine tumors and opens up for pretreatment assessment of the tumor proliferation rate. However, the FNB-yield was sparse, which in many cases lead to a falsely low estimation of the Ki-67 Index as compared with the Ki-67 Index in resection specimens. Thereby, the investigated approach resulted in a high rate of tumor under-grading with an apparent risk of incorrectly classifying true PanNET G2-tumors as G1-tumors at the preoperative stage.

Certainly, in non-functioning PanNETs, tumors of larger size have an increased likelihood of being of higher tumor grade as compared with smaller tumors^25^. Moreover, both MRI and EUS can determine tumor size with high precision^26^. Nevertheless, tumor size alone cannot act as a reliable surrogate marker of the tumor grade in PanNETs^27^. Therefore, an early and reliable estimation of the Ki-67 index in pretreatment PanNET tumor tissue would be highly valuable to estimate the tumor grade and thereby facilitate the decision on further clinical management and surgery.

According to the presented results, the 22-gauge reverse bevel FNB-needle seems to be appropriate in the EUS-based diagnosis of suspected PanNETs as such. The recorded sensitivity for PanNET of around 85% is comparable to findings in other studies investigating either the sensitivity of EUS-FNA^16,28^ or that of EUS-FNB^29,30^. In a very recent publication by Crino et al. including a high number of patients subjected to EUS-FNB (n = 231), the true diagnostic sensitivity of EUS-FNB cannot be estimated given the retrospective design of the study and the method applied for selection of cases to be included in the study^31^.

Relatively few studies have focused on factors with a potential impact on the sensitivity and the sampling yield of EUS-FNA/FNB in PanNET. We noticed a tendency, admittedly without a significant p-value, that EUS-FNB had a higher sensitivity in large (> 20 mm) tumors as compared with small (< 20 mm) tumors. Apart from that finding, we detected no factors associated with sufficient yield. In one study by Hijioka and colleagues, analyzing the yield of EUS-FNA, tumor location in the pancreatic head and heavy tumor fibrosis were found to be negative factors, while no other tested factors were associated with poor yield^32^. In our study, we recorded a numerically, but not significantly, higher rate of FNB-samples with a neoplastic cell count > 1000 cells in tumors located in the pancreatic head as compared with in the body-tail.

In the presented study cohort, the FNB-biopsy quality, i.e. the amount of neoplastic cells acquired from the target tumor, was often relatively poor. A vast majority of samples had a neoplastic cell count < 2000 cells and not few samples a cell count < 500 cells. It could be hypothesized that a high number of needle passes would increase the likelihood of a good FNB core with a high cell count. Although not statistically proven in our data set, we noticed a trend that that a higher number of FNB-needle passes lead to an increased rate of samples containing a minimum of 1000 neoplastic cells, Table 3. Accordingly, we suggest that endosonographers aim for at least 3 needle passes if using the reverse bevel needle in suspected PanNET. In the current study, gross examination of the FNB-core was performed to reassure adequate yield but also to avoid excessive needle passes. However, and according to a recent report, it might be that on-site evaluation of samples is of no significant benefit^33^.

The side-fenestrated FNB-needle has been shown to be significantly more often associated with a low cell count (< 500 cells) as compared with the end-cutting FNB-needles^31^. Indeed, during the last few years, there has been a growing body of scientific support in favor of end-cutting instead of side-fenestrated FNB-needles in^34–38^. Based on the results presented by us and by others in the aforementioned studies, we suggest and believe that the side-fenestrated design of the FNB-needle used in the current study is an important negative factor in the explanation of the imperfect FNB-yield. When the current study was designed, there was a lack of data and evidence on this topic. Since the endosonographers engaged in the current study had long experience of EUS, the competence of the endosonographer was much less likely a factor of importance.

Apparently, the Ki-67 Index in FNB-samples acquired with a 22-gauge side-fenestrated needle is unreliable. Multiple factors may account for this finding. First, in any sampling modality including EUS-FNB, there is an evident risk of sampling error irrespective of what technique or needle used, since tumor heterogeneity is significant and tumor hot spots with a high proliferation rate can be missed at sampling^39^. Grillo and co-workers estimated that it might be required to use a large 18 gauge-needle and the procurement of a 15 mm biopsy core to obtain a relatively reliable sample for grading of G2-tumor^40^. The acquisition of such a large sample by the use of EUS is quite demanding irrespective of the needle used. Second, the tumor microarchitecture of PanNETs could be non-favorable with respect to sampling via EUS-FNB. Third, and as hypothesized above, the construction and design of the reverse-bevel FNB-needle investigated in this study is likely less well adapted for sampling of PanNETs as compared with end-cutting EUS-FNB-needles^38^, which, most probably, should be used firsthand. Less likely, the experienced endosonographers engaged in the current study is an explanation for poor FNB-yield.

Significant efforts have been performed by others with the intent to assess the validity of pretreatment tumor grading of PanNETs by the use of samples acquired by EUS-guided sampling^41^. In a recent, retrospective publication, Leeds and colleagues concluded that EUS-FNB (n = 26) was superior to EUS-FNA (n = 35) in the diagnosis of PanNET. Still, the Ki-67 index assessed in EUS-FNB showed only a moderate, correlation (r = 0.65) with the Ki-67 Index in surgical specimens^42^. Consequently, the overall agreement was only moderate when comparing the tumor grade assessed in EUS-samples with the tumor grade in surgical specimens. Like in our study, several tumors [6/14 (43%)] classified as G2 in surgical specimens were classified as G1-tumors in EUS-FNB samples. Problematically, needles of various tip design (reverse bevel and fork-tip) and of various sizes (22/25 gauge) were used in the study by Leeds, which complicates the interpretation of the results and weakens the conclusions drawn by the authors. Moreover, two different classification systems for grading were used in parallel in the study.

Similarly, in a retrospective study analyzing 33 cases with PanNET Hwang et al. recorded that the Ki-67 Index in EUS-FNB samples was significantly lower than in the corresponding surgical specimens. The reverse bevel FNB-needle was used in the study but needles of various sizes (19/22/25 gauge) were used. In eight cases information on the needle size was lacking. The authors concluded that there was a substantial risk of under-grading in EUS-FNB samples of Grade 2 and Grade 3 PanNETs^43^. The very same conclusion was drawn in a small study including 10 patients and published in 2016^44^.

As an exception, in a retrospective study including 59 patients, Di Leo and co-workers reported a high tumor grading agreement (84%) comparing the Ki-67 Index in twenty-five EUS-FNB samples of various types of needles with that of available surgical specimens^45^. However, all EUS-FNB samples with a non-diagnostic yield was excluded in the above calculations making the true grading agreement most probably far lower than reported, at least if an intention to diagnose approach would have been applied. Moreover, in a majority of cases (80%) a 25-gauge EUS-FNA needle was actually used while a true 19/22-gauge EUS-FNB needle was used in only 20% of cases. Hence, in reality the above study could be considered a study rather analyzing the Ki-67 index in a mixed set of histopathology and cytopathology samples. Similarly, in the study by Kamata and colleagues, a grading concordance of 83% was reported comparing 25 gauge reverse bevel EUS-FNB with surgical specimens^46^. However, the concordance would drop significantly if an intention-to-diagnose analysis would have been performed instead of a per-protocol analysis. Paiella et al. suggested a relatively low risk of undergrading by EUS-sampling in 110 cases of PanNET, but again cases being non-diagnostic at EUS were excluded from the main analysis^47^.

In the study by Crino and co-workers mentioned above, the authors recorded a robust correlation between the Ki-67 Index in EUS-FNB samples and the Ki-67 Index in surgical specimens with only 3/77 (4%) cases being under-graded at EUS-FNB. However, in another 4 cases the Ki-67 Index could not be evaluated in the EUS-FNB samples, which thereby should be regarded as failures and incorrect grading. Interestingly, the authors also reported a close-to significant trend (p = 0.07) that the use of end-cutting FNB-needles (Fork-tip: n = 129; Franseen-tip: n = 24) was superior to the reverse-bevel FNB-needle (n = 78) in the acquisition of samples adequate for Ki-67-indexing. In contrast to the study by Crino and colleagues, we applied a prospective study design investigating one needle type only without any variation in needle size. Such a study design minimizes the number of confounding factors. Moreover, results become more easy to interpret, which in the current study equals clear support not to use the reverse bevel needle firsthand.

As in the above mentioned studies, we recorded a high degree of under-grading in EUS-FNB samples with several G2-tumors being assessed as G1-tumors. From a preoperative, management point of view, it might not be severely problematic if G2-tumors are falsely graded as G1-tumors since both groups are often candidates for surgery anyhow. Patients with a G1-tumor have a better prognosis, i.e. longer overall and disease free survival, compared with patients with a G2-tumor^48^. Nevertheless, the obviously quite sparse yield of reverse bevel EUS-FNB is indeed worrisome if to be used as a tool for the selection of patients for surgery among elderly patients or other patients with an estimated high risk–benefit of surgery in low grade tumors. The draw-back of most studies published on the topic, including the current one, is that very few study subjects harbor G3-tumors. Most probably, the reason for the lack of data in this group of patients is that only few patients with G3-tumors are referred for EUS as such since in many cases the preoperative diagnosis can be determined by other diagnostic modalities.

Others have evaluated digital quantification of Ki-67 positive cells in various neoplasms, for example in pulmonary NETs^11^ and in PanNETs^10^. However, in a majority of publications including both the mentioned ones, quantification has been performed in resection specimens and not in pretreatment tumor tissue. In the study by di Leo et al. manual quantification of the Ki-67 positive nuclei in FNB samples was performed^45^ as was the case in the study by Leeds and co-workers^42^. In the retrospective study by Hwang and colleagues, a digital image analyzer was indeed applied for cell counting, but the software used was different from the one used in the current work.

It could be discussed, how many groups of cohesive cells acquired by EUS-FNB that should be included in the calculation of the Ki-67 Index and the tumor grade of EUS-FNB. In the current study we decided to include the three largest groups of neoplastic cells. To rule out this cut-off level as a source of bias, we performed the very same calculations of the Ki-67-index also in in the ten largest groups of cohesive cells, without any significant difference in outcome, Supplementary Material. Apparently, the inclusion of as much as ten groups of neoplastic cells did not reduce the rate of under-grading.

What about using EUS-FNA samples and smears for the preoperative assessment of the Ki-67 Index? In a meta-analysis published in 2016 including thirteen studies (263 cases), the pooled sensitivity of the Ki-67 Index estimated in EUS-FNA smears was 64% in discriminating G1 from G2/G3-tumors^49^. In a selected group of patients (n = 15), the assessment of the Ki-67 Index in cellblocks preparations of EUS-FNA aspirates was reported to be a promising alternative to EUS-FNB reaching an impressive 100% agreement with the Ki-67 Index of surgical specimens^50^. However, all cases with an EUS-FNA cell count of < 400 cells were excluded in the analysis. Most probably, the inclusion of these excluded cases in an intention-to-diagnose analysis would have lowered the agreement rate. The aim of the current study was not to evaluate EUS-FNA-samples for the estimation of the Ki-67-index. Nevertheless, in the patients subjected to EUS-FNA at our center during 2015–2019, the cytopathologist reported an acceptable amount of tumor cells for assessment of the Ki-67 Index in only somewhat more than half of the patients.

To the best of our knowledge, the current work is the first large prospective study analyzing the accuracy of Ki-67-indexing in EUS-FNB specimens for pretreatment grading of PanNETs. The study is also strengthened by the fact that no case was lost from follow-up. The use of the identical WHO classification system throughout the study is another advantage, which guarantees reliability of the results and facilitates the interpretation of the results. Finally, the study pathologist was blinded to the Ki-67-calculations both in surgical specimens and in EUS-FNB samples.

There are some limitations of the study. This was a single-center study, which accounts for consistency in the method used. On the other hand, the number of resected was relatively small and the results presented need to be validated in external centers. Many PanNET-patients, especially older patients with small tumors, were not subjected to surgery but instead surveillance. Therefore, and quite obviously, the Ki-67 Index of all FNB-samples could not be compared with that of corresponding surgical specimens. A potential weakness is the fact that not all eligible patients were subjected to EUS-FNB but rather to EUS-FNA because of the reasons mentioned above. A final limitation is the fact that no fixed number of FNB-passes was performed in the study. Instead, the endosonographer based the number of passes on the macroscopic yield of the FNB-core.

In conclusion, EUS-guided fine needle biopsy sampling performed with a 22-gauge reverse bevel needle is sensitive for the diagnosis of PanNET. However, the biopsy yield is sparse and the quality of the biopsy is inadequate for reliable Ki-67-indexing and grading of tumors. Most probably, that risk is at least partially due to the small size of tissue cores gained by EUS-FNB. Therefore, the studied EUS-FNB approach leads to a significant risk for under-grading of PanNETs. Not unlikely, end-cutting FNB-needles might be better options with a higher likelihood for correct tumor grading. Improvement in needle design and optimization of sampling technique is still warranted and further studies on the topic are needed.

## Supplementary Information

Supplementary Information.

## Data Availability

Data will be available upon request.

## Abbreviations

EUS: Endoscopic ultrasound
FFPE: Formalin-fixed paraffin embedded
FNA: Fine-needle aspiration
FNB: Fine-needle biopsy
