# Supplementary material for: Endoscopic ultrasound-guided side-fenestrated needle biopsy sampling is sensitive for pancreatic neuroendocrine tumors but inadequate for tumor grading: a prospective study
Source: Sci Rep. 2022 Apr 8;12:5971. doi: 10.1038/s41598-022-09923-1 (PMC8993931; doi:10.1038/s41598-022-09923-1)
Supplement: Supplementary file 1 — Supplementary Information. [file 41598_2022_9923_MOESM1_ESM.docx]

**Supplementary Material**

As described in the Methods section, the biopsy quality, i.e. the cell count in EUS-FNB samples, was assessed not only in the three largest groups of cohesive cells identified but also in the ten largest groups of cohesive cells identified. Likewise, the pretreatment Ki-67-index (Ki-67_EUS_) and the tumor grade (GRADE_EUS_) was estimated.

Here, we present the outcome parameters based on the calculations performed in the ten largest groups of cohesive cells:

***The biopsy quality and neoplastic cell count in EUS-FNB samples***

The median neoplastic cell count of the ten largest group of cohesive neoplastic cells was 1644 (IQR: 777–5079) with 38/42 (90 %), 29/42 (69 %), and 19/42 (45 %) cases exceeding 500, 1000, and 2000 neoplastic cells respectively.

***Accuracy of Ki-67-indexing and grading of PanNET in EUS-FNB samples***

In the 17 cases which were subjected to surgery (#34 not included due to staining artifacts), there was only a moderate correlation comparing the Ki-67_EUS_ and the Ki-67_SURG_ (Pearson r=0.65, r^2^=0.42, p=0.005).

Based on the estimated Ki-67_EUS_, the GRADE_EUS_ was found to have a weak level of agreement (κ=0.14) in an intention-to-treat analysis (including non-diagnostic FNB-samples) and weak (κ=0.26) in a per-protocol analysis (excluding non-diagnostic FNB-samples). In the intention-to-treat analysis, only 3/12 (25%) tumors graded as G2 in surgical specimens (GRADE_SURG_) were indeed correctly graded as G2 also in EUS-FNB samples (GRADE_EUS_). Analyzing only the cases with a Cell Count_EUS_ >1000 cells (n=12: G1 n=7; G2 n=5), still 4/5 (80%) G2-tumors (GRADE_SURG_) were graded as G1-tumors in FNB biopsy samples (GRADE_EUS_), **Supplementary Table 1**.

**Supplementary Table 1 The tumor grade**^a^ **of resected PanNETs (n=22) as assessed in EUS-FNB samples (GRADE_EUS_) and in the corresponding surgical specimens (GRADE_SURG_)**

| **GRADE_EUS_** | **GRADE_SURG_** | | |
| --- | --- | --- | --- |
|  | G1 | G2 | G3 |
| G1 | 7 | 7 | 0 |
| G2 | 0 | 3 | 0 |
| G3 | 0 | 0 | 0 |
| Non-diagnositc FNB^b^ | 3 | 2 | 0 |

^a)^ Grading based on the assessment of the Ki-67 Index in the ten largest group of cohesive neoplastic cells

^b)^ EUS-FNB samples being non-diagnostic at pathology
